# Urinary orosomucoid and retinol binding protein levels as early diagnostic markers for diabetic kidney Disease

**DOI:** 10.1186/s12882-023-03363-3

**Published:** 2023-12-04

**Authors:** Xue-hong Zhou, Shu-yan Liu, Bo Yang, Yong-feng Li, Bao-hua Hou

**Affiliations:** 1 Department of Endocrinology, The First Affiliated Hospital of Henan Polytechnic University (Jiaozuo Second People’s Hospital), Jiaozuo, China; 2 Department of Neurology, The First Affiliated Hospital of Henan Polytechnic University (Jiaozuo Second People’s Hospital), Jiaozuo, China; 3 College of Medicine, Henan Polytechnic University, Jiaozuo, China; 4 Central Laboratory, The First Affiliated Hospital of Henan Polytechnic University (Jiaozuo Second People’s Hospital), Jiaozuo, China

**Keywords:** Diabetic Nephropathy, Diagnosis, Urine microalbumin, Retinol-binding protein

## Abstract

**Background:**

Diabetic kidney disease (DKD) is the most common microvascular complication of diabetes, which has been a major cause of end-stage renal failure. Diagnosing diabetic kidney disease is important to prevent long-term kidney damage and determine the prognosis of patients with diabetes. In this study, we investigated the clinical significance of combined detection of urine orosomucoid and retinol-binding protein for early diagnosis of diabetic kidney disease.

**Methods:**

We recruited 72 newly diagnosed patients with type 2 diabetes and 34 healthy persons from August 2016 to July 2018 at the First Affiliated Hospital of Henan Polytechnic University (Jiaozuo Second People’s Hospital). Using the Mogensen grading criteria, participants were classified as having diabetes or diabetic kidney disease, and healthy persons constituted the control group. Urine orosomucoid and retinol-binding protein levels were measured and correlated with other variables.

**Results:**

With the aggravation of renal damage, the level of urinary mucoid protein gradually increased. Urinary retinol-binding protein and microalbumin levels were significantly higher in the diabetes group than in control and nephropathy groups. Orosomucoid and retinol-binding protein might be independent risk factors for diabetes and diabetic kidney disease. Urinary orosomucoid significantly correlated with retinol-binding protein and microalbumin levels in the diabetic kidney disease group.

**Conclusion:**

Elevated urine orosomucoid and retinol-binding protein levels can be detected in the early stages of type 2 diabetic kidney disease. Both of these markers are important for diabetic kidney disease detection and early treatment.

## Introduction

Diabetic kidney disease (DKD) is the most common and severe chronic vascular complication among patients with type 2 diabetes mellitus (T2DM) [1]. It leads to chronic renal failure and is the leading cause of death due to diabetes. However, DKD patients have no obvious symptoms in the early stage. Common diagnostic indicators of DKD include 24-hour urine microalbumin (MAL), urea nitrogen, and serum creatinine levels. However, they can be affected by many factors, such as urinary tract or systemic infections, strenuous exercise, bleeding, or drugs that affect the kidneys [2]. The accuracy and specificity of these indicators are not high, and they have limitations [3]; thus, more research is needed to identify newer, more accurate, and specific early diagnostic markers of DN. Presently, preliminary progress has been made in kidney disease research using proteomics technology. Diabetic urine proteome research has shown that some protein markers have a predictive effect on early DKD [4, 5]. Screening for differentially expressed proteins in the urine of DKD patients, performing mass spectrometry and bioinformatics analysis of the differential proteins, and selecting and obtaining six differential proteins such as orosomucoid and retinol-binding protein (RBP) for research will help in the early diagnosis of DKD. El-Beblawy Nagham MS assessed serum and urinary orosomucoid levels in children and adolescents with type 1 diabetes and suggested that orosomucoid is a significant independent factor for diabetic microvascular complications and can be considered as an early marker of renal injury [6]. Wang evaluated the value of urinary orosomucoid in early renal impairment screening in T2DM patients and found orosomucoid level had high diagnostic efficiency to aid in the early detection of renal impairment in T2DM patients [7]. Mahfouz suggested that the RBP4 marker may serve as a tool to follow-up on the development and progression of DKD [8]. In this study, the urine orosomucoid and RBP levels were measured in healthy people, patients with T2DM, and patients with early DKD. The differences between the three groups were compared. We also assessed their clinical significance in the diagnosis of early type 2 DKD and their clinical value in the progression of nephropathy.

## Methods

### Study subjects

Thirty-four healthy people who underwent physical examination at the First Affiliated Hospital of Henan Polytechnic University (Jiaozuo Second People’s Hospital) between August 2016 and July 2018 were categorized as the normal control (NC) group, including 18 males and 16 females, with an average age of 47.9 ± 14.2 years. Seventy-seven patients with T2DM who were hospitalized at the same time were assessed according to the Mogensen classification criteria for the degree of kidney damage. We recorded the medical history and complications of all patients. Complications of diabetes include DKD and diabetic retinopathy (DR). These patients were categorized as those with T2DM (T2DM group; n = 38; microalbumin (MAL) < 30 mg/24 h), which included 21 males and 17 females, with an average age of 48.7 ± 13.6 years, and patients with early DKD and combined DR (T2DKD group; n = 34; MAL 30–300 mg/24 h), which included 19 males and 15 females, with an average age of 49.1 ± 14.4 years. Diabetes was diagnosed and classified according to the 1999 diagnostic criteria of the World Health Organization [9]. The exclusion criteria were diabetic ketosis, hyperglycemia and osmotic pressure syndrome, combined fever and infection, acute cardiocerebrovascular diseases and urinary system diseases (kidney stones, acute and chronic nephritis, and nephrotic syndrome), non-diabetic congestive heart failure, liver dysfunction, rheumatic diseases, hematological diseases, pregnancy, tumors, fractures, primary hyperparathyroidism, a history of kidney transplant and intake of glucocorticoids, history of immunosuppressant and nephrotoxic drugs use, history of renal damage caused by strenuous exercise, and severe hypertension.

### Data collection

We recorded the medical history and complications of all patients and measured their diastolic blood pressure (DBP), systolic blood pressure (SBP), height (cm), and weight (kg). The levels of total cholesterol (CHOL), low-density lipoprotein cholesterol (LDL), triglycerides (TG), fasting blood glucose (FBG), 2-hour blood glucose, serum creatinine (SCr) and urine orosomucoid, and RBP were measured using Applied blood biochemical detector (Hitachi 7600, Hitachi, Japan). HLC-723 HbA1c Analyzer (Japan Toshiba, Tokyo, Japan), i.e., high-pressure liquid phase ion-exchange chromatography was used to determine glycosylated hemoglobin (HbA1c) levels. The urine specimen was collected between 10 pm and 6 am, and the total urine volume (mL) was recorded after mixing. IMMAGE 800 protein chemistry analyzer (Beckman Coulter, USA) was used to detect urine MAL. The urine orosomucoid level was determined using the rate scattering immunoturbidimetric method performed on the Array 360 System (Beckman Coulter, USA). Orosomucoid reagents, calibration cards, and calibration were provided by the manufacturer. The detection temperature was 37℃, while the measurement wavelength of the instrument was 340 nm; the immunoturbidimetric measurement was performed after calibration. CV within and between batches was less than 5%. The CKD-EPI equation [10] was used to estimate the glomerular filtration rate (eGFR).

### Statistical analysis

For normally distributed data determined using the Shapiro–Wilk’s test, the indicators in each group were expressed as the mean ± standard deviation. The chi-square test was used to compare quantitative data between groups. The mean values for each of the three groups were compared using one-way analysis of variance. If there were significant differences between the groups, intra-group comparisons were performed using the least significant difference. A binary logistic regression model was used to determine the factors associated with T2DM and T2DKD, and correlation analyses were performed using Spearman’s rank correlation. The receiver operating characteristic (ROC) curve was used to analyze the diagnostic points and diagnostic value of orosomucoid and RBP in DKD. All statistical analyses were performed using SPSS version 23.0 (IBM Corp., Armonk, NY, USA). A two-tailed test with P < 0.05 was considered significant.

## Results

There were no significant differences in age and gender among the three groups (P > 0.05). There were no significant differences in DBP, BMI, CHOL, TG, and LDL levels among the three groups (P > 0.05) (Table 1).

**Table 1:** Comparison of general clinical data among the three groups

Groups	NC (n = 34)	T2DM (n = 38)	T2DKD (n = 34)	F	P
BMI, kg/m2	26.19 ± 1.28	26.32 ± 1.31	26.27 ± 1.24	0.094	0.911
SBP, mmHg	117.82 ± 12.06	130.61 ± 12.44a	142.55 ± 14.78ab	33.11	< 0.001
DBP, mmHg	80.48 ± 9.16	83.26 ± 9.36	85.19 ± 10.08	2.098	0.128
HbA1c (%)	5.3 ± 0.51	10.45 ± 2.65a	10.27 ± 2.77a	58.55	< 0.001
FBG, mmol/L	4.63 ± 0.52	9.98 ± 4.63a	10.69 ± 4.27a	27.710	< 0.001
CHOL, mmol/L	4.35 ± 0.66	4.82 ± 1.08	4.83 ± 1.14	2.670	0.074
TG, mmol/L	1.29 ± 0.54	1.3 ± 0.61	1.33 ± 0.56	0.045	0.956
LDL, mmol/L	2.17 ± 0.43	2.33 ± 0.61	2.41 ± 0.69	1.474	0.234

^a^Compared with NC: P < 0.05,

^b^Compared with T2DM: P < 0.05

Abbreviations: BMI, body mass index; SBP, systolic blood pressure; DBP, diastolic blood pressure; HbA1c (%), glycosylated hemoglobin; FBG, mmol/L, fasting blood glucose; CHOL, mmol/L, cholesterol; TG, mmol/L, triglycerides; LDL, mmol/L, low-density lipoproteins

There were significant differences in SBP, HbA1c, FBG between the NC and the other two groups (P < 0.05). However, there was no significant difference in the general clinical data between the T2DM and T2DKD groups (P > 0.05). As renal damage increased in patients, urine orosomucoid levels gradually increased as well (P < 0.05) (Table 1).

Urine RBP and MAL levels in the T2DKD group were significantly higher than those in the NC and T2DKD groups (P < 0.001). The eGFR levels in the T2DKD group were significantly lower than those in the NC and T2DM groups (P < 0.001). There were no significant differences in RBP, eGFR, and MAL levels between the NC and T2DM groups (P > 0.05) (Table 2).

**Table 2:** Comparison of urinary orosomucoid, RBP, MAL, and eGFR levels among the three groups

Groups	NC (n = 34)	T2DM (n = 38)	T2DKD (n = 34)	F	P
Orosomucoid, mg/L	9.45 ± 2.03	18.35 ± 4.04^a^	29.46 ± 6.13 ^ab^	177.82	< 0.001
RBP, mg/L	0.26 ± 0.07	0.31 ± 0.09	0.95 ± 0.28 ^ab^	172.56	< 0.001
eGFR, mL/min/1.73 m^2^	108.08 ± 13.73	102.17 ± 10.12	94.92 ± 10.57^ab^	11.11	< 0.001
MAL, mg/24 h	10.22 ± 6.42	12.13 ± 7.83	199.65 ± 49.72^ab^	492.19	< 0.001

^a^Compared with NC group: P < 0.05

^b^Compared with T2DM group: P < 0.05

Abbreviations, RBF, renal blood flow; eGFR, estimated glomerular filtration rate; MAL, microalbumin

For the NC group and T2DM group, the dependent variable was whether T2DM had occurred (Yes = 1, No = 0) and the independent variables were the four variables (SBP, HbA1c, FBG, and orosomucoid) with differences between the two groups, as shown in Tables 1 and 2. A binary logistic regression model was established and used to determine the influence of these four variables on T2DM (Table 3), and all were shown to be risk factors (all OR > 1, p < 0.05).

**Table 3:** Classification of clinical, biochemical parameters and biomarkers (RBP, Orosomucoid) in T2DKD as per micro albumin tertiles

Variable	Regression coefficient (B)	Significance level (P)	Odds ratio (OR)	95% CI of the OR	
				Lower limit	Upper limit
SBP, mmHg	0.106	0.0001	1.112	1.056	1.170
HbA1c (%)	2.133	0.001	8.438	2.320	30.688
FBG, mmol/L	1.022	0.0001	2.779	1.663	4.646
Orosomucoid, mg/L	0.964	0.001	2.621	1.521	4.516

Abbreviations: SBP, systolic blood pressure; HbA1c, glycosylated hemoglobin; FBG, fasting blood glucose.; OR, odds ration; CI, confidence interval

For the T2DM group and T2DKD group, the dependent variable was whether T2DKD had occurred (Yes = 1, No = 0) and the independent variables were the five variables with differences between the two groups, as shown in Tables 1 and 2. A binary logistic regression model was established for analysis (Table 4).

**Table 4:** Binary logistic regression analysis of the factors associated with Type 2 diabetic kidney disease

Variable	Regression coefficient (B)	Significance level (P)	Odds ratio (OR)	95% CI of the OR	
				Lower limit	Upper limit
SBP, mmHg	0.089	0.0001	1.093	1.045	1.143
Orosomucoid, mg/L	0.626	0.0001	1.871	1.360	2.574
RBP, mg/L	0.241	0.023	13.305	9.079	26.000
eGFR, mL/min/1.73 m^2^	-0.054	0.021	0.948	0.905	0.992
MAL, mg/24 h	0.892	0.001	2.441	1.070	3.149

Abbreviations: RBF, renal blood flow; eGFR, estimated glomerular filtration rate; MAL, microalbumin; SBP, systolic blood pressure; OR, odds ration; CI, confidence interval

Of the five factors that were included in the regression model (p < 0.05), SBP, orosomucoid, RBP, and MAL were all determined to be risk factors (OR > 1), and eGFR was shown to be a protective factor (OR = 0.948 > 1). Correlation analysis showed that in the T2DKD group, the urinary orosomucoid level was significantly positively correlated with RBP (*r* = 0.489) and MAL (*r* = 0.513). RBP and MAL were significantly positively correlated with a correlation coefficient of 0.468. eGFR and urine orosomucoid, RBP, and MAL were significantly negatively correlated (*r* = -0.577, -0.474, and − 0.466, respectively).

**Fig. 1:**
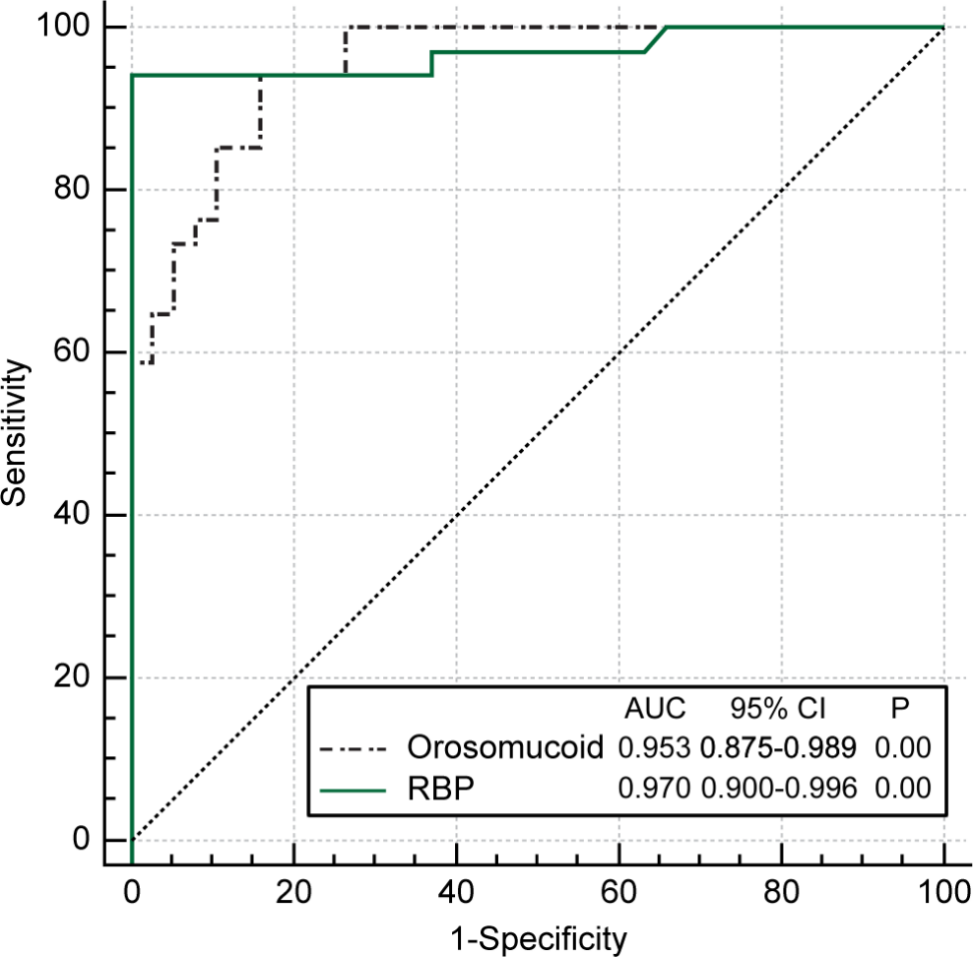
ROC curve analysis was used to assess the diagnostic points and diagnostic value of orosomucoid and that of RBP to predict DKD.

ROC curve analysis was used to assess the diagnostic points and diagnostic value of orosomucoid and that of RBP to predict DKD. Figure 1; Table 5 show the areas under the ROC curves for orosomucoid and RBP with the respective standard error values.

**Table 5:** Areas under the two ROC curves for predicting diabetic kidney disease

Variable	Area under the ROC curve	Standard error	P	95% confidence interval	
				-LR	+LR
Orosomucoid	0.953	0.021	0.00	0.875	0.989
RBP	0.970	0.022	0.00	0.900	0.996

Abbreviations: RBP, renal blood flow; ROC, receiver operating characteristic; -LR, negative likelihood ratio; +LR, positive likelihood ratio

The diagnostic value of DKD had improved; however, no significance was observed (*Z* = 0.598, P = 0.550 > 0.05). The diagnostic point of orosomucoid was 22.43, sensitivity was 0.941, specificity was 0.842, and Youden’s index was 0.783. The diagnostic point of RBP was 0.53, sensitivity was 0.942, specificity was 1.000, and Youden’s index was 0.941.

## Discussion

DKD has become the leading indication for dialysis due to end-stage renal disease (ESRD) [11]. Recent findings suggest that immune-mediated inflammatory processes play a crucial role in DKD. Many pre-inflammatory cells, growth regulators, and adhesion factors interact with each other and cross-link, resulting in expanding the corresponding cascade of inflammation [12]. In recent years, the rapid development of proteomics technology has provided us with new methods and ideas for identifying early diagnostic markers of DKD. Proteomics techniques have been used to identify disease-specific biomarkers and other related proteins in urine. Differential proteins have been identified, and some protein markers were found to have predictive effects on glomerular diseases.

Orosomucoid protein is a non-specific acute phase reaction protein that is mainly synthesized and secreted by the liver; it is low in healthy body fluids but is significantly increased in a state of inflammation or during tumor growth. Orosomucoid can act in damaged areas, be released into the circulation and intercellular fluid, and become involved in the induction and regulation of body damage, immune, and inflammatory responses [13]. Elevated urine orosomucoid levels in T2DM patients have predictive effects on cardiovascular complications and mortality [14]. El-Beblawy et al. [15] pointed out that orosomucoid is an independent factor for diabetic microvascular complications and can be considered an early marker of kidney damage. Fandiño-Vaquero et al. [16] also found that orosomucoid levels increased in patients with T2DM and might mirror local endothelial dysfunction or inflammatory processes. Although there are existing studies on orosomucoid, studies on changes in orosomucoid concentration in urine during the early stage of DKD is lacking. In this study, we found that as the disease progressed, urine orosomucoid levels gradually increased (P < 0.001). Orosomucoid might be an independent risk factor for T2DM and T2DKD, and it had a significant positive correlation with MAL (*r* = 0.489) and a significant negative correlation with eGFR (*r* = -0.577). The results also revealed an increase in orosomucoid in the early stage of DKD, suggesting that this increase may promote the occurrence and development of DKD.

RBP is filtered through the glomerulus and absorbed and degraded by proximal tubular epithelial cells. Therefore, it is generally stable in urine, difficult to decompose, and has a low excretion rate. An increase in RBP excretion may reflect the extent of damage [17, 18]. Studies have shown that urine RBP levels in patients with T2DM are closely related to DKD [19, 20]. Some studies have shown that urine RBP can be used to assess the degree of renal interstitial fibrosis due to various causes, progress with ESRD dialysis, and even diabetes related to an increased risk of death [21, 22]. This study found that urine RBP and MAL levels in the T2DKD group were significantly increased (P < 0.001). Therefore, RBP might be an independent risk factor for T2DKD, owing to a significant positive correlation with MAL (*r* = 0.468) and a significant negative correlation with eGFR (*r* = -0.474). Urine RBP may reflect early renal damage in DKD. In the area under the ROC curve for predicting DKD using orosomucoid and RBP, both factors had high sensitivity and specificity. Therefore, both orosomucoid and RBP can be used to diagnose DKD.

This study’s main limitation is that all the participants were residents of Henan Province, China, and the sample size was small. Further verification is needed through large sample-size studies and multicenter studies.

## Conclusions

Urine orosomucoid and RBP can serve as potential markers for the early diagnosis of DKD, facilitating timely treatment. However, the underlying molecular mechanisms and the clinically important levels of these potential biomarkers need to be studied further.

## Data Availability

The datasets used and analyzed during the current study are available from the corresponding author on reasonable request and with the permission of The First Affiliated Hospital of Henan Polytechnic University ethics committee.

## Abbreviations

AKI: Acute Kidney Injury
BMI: Body mass index
CHOL: Cholesterol
CI: Confidence interval
DBP: Diastolic blood pressure
DKD: diabetic kidney disease
ESRD: End-stage renal disease
FBG: Fasting blood glucose
LDL: Low-density lipoprotein
MAL: Microalbumin
NC: Normal control
NGAL: Neutrophil gelatinase-associated lipocalin
RBF: Renal blood flow
RBP: Retinol binding protein
ROC: Receiver operating characteristic
SBP: Systolic blood pressure
